# Evidence that faecal carriage of resistant *Escherichia coli* by 16-week-old dogs in the United Kingdom is associated with raw feeding

**DOI:** 10.1016/j.onehlt.2022.100370

**Published:** 2022-01-15

**Authors:** Oliver Mounsey, Kezia Wareham, Ashley Hammond, Jacqueline Findlay, Virginia C. Gould, Katy Morley, Tristan A. Cogan, Katy M.E. Turner, Matthew B. Avison, Kristen K. Reyher

**Affiliations:** a University of Bristol School of Cellular & Molecular Medicine, Biomedical Sciences Building, University Walk, Bristol BS8 1TD, United Kingdom; b University of Bristol Veterinary School, Langford House, Langford, Bristol BS40 5DU, United Kingdom; c University of Bristol Medical School, Population Health Sciences, Canynge Hall, 39 Whatley Road, Bristol BS8 2PS, United Kingdom

**Keywords:** Fluoroquinolone, Zoonosis, Genomics, Urinary tract infection

## Abstract

We report a survey (August 2017 to March 2018) and risk factor analysis of faecal carriage of antibacterial-resistant (ABR) *Escherichia coli* in 223 16-week-old dogs in the United Kingdom. Raw feeding was associated with the presence of fluoroquinolone-resistant (FQ-R) *E. coli* and those resistant to tetracycline, amoxicillin, and streptomycin, but not to cefalexin. Whole genome sequencing of 36 FQ-R *E. coli* isolates showed a wide range of sequence types (STs), with almost exclusively mutational FQ-R dominated by ST744 and ST162. Comparisons between *E. coli* isolates from puppies known to be located within a 50 × 50 km region with those isolated from human urinary tract infections (isolated in parallel in the same region) identified an ST744 FQ-R lineage that was carried by one puppy and caused one urinary tract infection. Accordingly, we conclude that raw feeding is associated with carriage of ABR *E. coli* in dogs even at 16 weeks of age and that bacteria carried by puppies are shared with humans. We therefore suggest that those who feed their dogs raw meat seriously consider the potential ABR-transmission threat their pet may become as a result and deploy appropriate hygiene practices in mitigation.

## 1 Introduction

Antimicrobial resistance and particularly antibacterial resistance (ABR) has many negative impacts on the health and welfare of humans and animals including increased morbidity and mortality and an increase in treatment costs [1]*.* ABR is linked across human populations, animal populations and the environment [2]. A key ABR pathogen of relevance is *Escherichia coli*, which is carried in the intestines of humans as well as farmed and companion animals, and causes a significant disease burden in all three, and especially in humans [3].

Several recent studies using whole genome sequencing (WGS) have concluded that sharing of ABR bacteria between farmed animals and humans is uncommon in the United Kingdom [[4], [5], [6], [7], [8]]. However, ABR bacteria found in domestic pet dogs and their owners are often indistinguishable [[9], [10], [11], [12], [13]].

There are several ways that domestic pet dogs may become colonised by ABR *E. coli* and so bring them into the home. Ingestion is an essential part of colonisation; therefore, ingestion of faeces or faecally contaminated food or water by dogs may be a key source of ABR bacteria derived from humans and farmed animals [14,15]. Research has also suggested that dogs become colonised by ABR bacteria when visiting veterinary hospitals [16,17] and that antibacterial exposure makes colonisation more likely [18]. Associations have also been found between increased carriage of ABR bacteria following coprophagia and with the feeding of raw poultry [[19], [20], [21]]. Of direct relevance to the present study, two UK studies have identified associations between ABR in faecal *E. coli* of adult dogs and those dogs being fed raw meat [19,21].

Up to now, there has not been any published work reporting very early life risk factors for carriage of ABR *E. coli* in domestic pet dogs. In the UK, current recommendations are for juvenile dogs to be weaned onto solid food at six to eight weeks of age and to stay with their mother until eight weeks of age. Owners are usually advised not to walk their dog outside in public places until approximately 12 weeks of age when the dog is fully vaccinated [22].

In this study, risk factors were investigated to explore associations between various lifestyle factors and the detection of ABR *E. coli* in faecal samples taken from dogs at 16 weeks of age. Practices and behaviours that might increase ingestion of faecal bacteria from the environment or food were particularly considered. Furthermore, WGS was used to characterise fluoroquinolone resistant (FQ-R) *E. coli* carried by these dogs and phylogenetic analysis was used to compare these isolates with others causing urinary tract infections (UTIs) in humans living in the same geographical region as the dogs.

## 2 Materials and methods

### 2.1 Recruitment of the cohorts

In total, 295 dog owners were recruited to take part in this study in two ways: (i) 236 were already recruited to the Dogs Trust “Generation Pup” project, a longitudinal study examining the health, welfare and behaviour of dogs across the UK [23] and (ii) 59 were locally recruited via word-of-mouth advertisement to clients bringing young dogs in for routine checks to veterinary practices in Somerset and Bristol, via puppy socialisation classes and via social media as well as local media advertisement. Locally recruited owners answered survey questions (listed in Table 1). As part of Generation Pup, owners completed more extensive surveys relating to their dogs at 16 weeks of age and responses to relevant survey questions (Table 1) were extracted from wider Generation Pup survey data. Questions were worded identically in the two surveys to ensure comparability. All dog owners also supplied a single faecal sample from their dog at 16 weeks of age, collected from the ground after defecation. Only one dog was included in the study from each household. The only other exclusion criterion was for dogs that had been previously hospitalised.

**Table 1:** Baseline data for all 16-week-old dogs (*n* = 223) and associations with risk factors for carriage of *E. coli* resistant to at least one test antibacterial. *p*-values were calculated using the Pearson Chi-squared test (Stata/IC 15.1, StataCorp LLC, College Station, TX, USA).

Risk factor from questionnaire	Response to question	Response to question total (*n* = 223)	Also resistant to at least one antibiotic (*n* = 106)	*p*-value
Fed raw food	Yes	43	32/43	<0.001
	No	180	76/180	
Walked in town	Yes	181	84/181	0.21
	No	42	24/42	
Walked on farmland	Yes	142	69/142	0.95
	No	81	39/81	
Walked on beaches	Yes	103	52/103	0.57
	No	120	56/120	
Walked in the countryside	Yes	191	95/191	0.34
	No	32	13/32	
Walking near cattle	Yes	84	37/70	0.31
	No	139	71/139	
Swum/paddled/played in salt water	Yes	62	32/62	0.56
	No	161	76/161	
Swum/paddled/played in lake water	Yes	29	17/29	0.24
	No	194	91/194	
Swum/paddled/played in river water	Yes	66	33/66	0.76
	No	157	75/157	
Swum/paddled/played in pond water	Yes	65	38/65	0.06
	No	158	70/158	

### 2.2 Ethical approvals

All dog owners were recruited between August 2017 and March 2018, and all owners gave consent. Ethical approval for this study was granted both by the Animal Welfare and Ethics Review Body (UB/17/053) and University of Bristol Health Sciences Student Research Ethics Committee (56783).

### 2.3 Faecal samples and processing

All dog owners were supplied with a sample collection pack comprised of a specimen bottle, gloves, biohazard bag and a freepost envelope. Faecal samples were sent by post to the University of Bristol's Veterinary School alongside the consent form and, for locally recruited dogs, a questionnaire. Were recruited and data for 223 dogs were included in the analysis. Of 295 dog owners recruited, 14 did not return a fully completed questionnaire, so they were excluded from further analysis. To process each faecal sample, approximately 0.1–0.5 g of faeces was taken and weighed. Ten millilitres per gram of phosphate buffered saline (PBS) was added to the sample and the mixture vortexed. Next, 0.5 mL of the faecal/PBS homogenate was added to 0.5 mL of 50% *v*/v sterile glycerol and processed as below.

### 2.4 Testing for ABR bacteria

Data were collapsed into a binary “positive/negative” outcome for the homogenate derived from each faecal sample. ABR positivity was defined by the appearance (following 37°C overnight incubation) of blue/green *E. coli* colonies after spreading 20 μL of faecal homogenate (or a 10-fold dilution in PBS if inoculum effect was observed) onto Tryptone Bile X-Glucuronide (TBX) agar plates containing either 0.5 mg/L ciprofloxacin (to identify FQ-R), 16 mg/L cefalexin, 8 mg/L amoxicillin, 16 mg/L tetracycline, or 64 mg/L streptomycin. These concentrations were chosen based on relevant human clinical breakpoints as defined by the European Committee on Antimicrobial Susceptibility Testing [24]. Faecal homogenates were also plated onto non-antibiotic TBX agar and samples were only included in the study if ≥10 *E. coli* cfu/μL were detected in an undiluted faecal homogenate. Therefore, the limit of detection for ABR for all faecal homogenates included in the analysis was ≤0.5% prevalence. Of 295 dog owners recruited, 58 faecal samples were excluded based on this criterion.

### 2.5 Risk factor analysis

Univariable and multivariable logistic regression models were used to evaluate associations between ABR *E. coli* positivity in homogenates derived from faecal samples and risk factors identified from the survey data (Stata/IC 15.1, StataCorp LLC, College Station, TX, USA). A backward stepwise method was used. In this method the full set of possible factors was analysed, with the least significant factors removed one at a time until all remaining factors had *p-*values of 0.05 or less. For the risk factor analysis, questionnaire answers were collapsed into binary ‘Yes/No’ variables; questionnaire answers of ‘sometimes’, ‘often’, ‘almost always’ and ‘frequently’ were all categorised as ‘Yes’.

### 2.6 Isolates from human infections

WGS data for 188 FQ-R human urinary *E. coli* from a 50 × 50 km region (including the homes of the 59 locally recruited dogs collected during the same timespan as the collection of faecal samples from these puppies) has been reported previously [8].

### 2.7 WGS analysis of FQ-R E. coli

WGS of deduplicated, representative FQ-R isolates from puppies was performed by MicrobesNG (https://microbesng.uk/) on a HiSeq 2500 instrument (Illumina, San Diego, CA, USA) using 2 × 250 bp paired end reads. Reads were trimmed using Trimmomatic [25] and assembled into contigs using SPAdes [26] 3.13.0 (http://cab.spbu.ru/software/spades/). Contigs were annotated using Prokka [27]. ABR genes were assigned using the ResFinder [28] and Sequence Types designated by MLST 2.0 [29] on the Centre for Genomic Epidemiology (http://www.genomicepidemiology.org/) platform. Single nucleotide polymorphism (SNP) distance analysis was performed using SNP-dists (https://github.com/tseemann/snp-dists).

### 2.8 Phylogenetic analysis

Sequence alignment and phylogenetic analysis was carried out using the Bioconda channel [30] on a server hosted by the Cloud Infrastructure for Microbial Bioinformatics [31]. The reference sequence was *E. coli* ST131 isolate EC958 complete genome (accession: HG941718). Sequences were first aligned to a closed reference sequence and analysed for SNP differences, whilst omitting insertion and deletion elements, using the Snippy alignment program (https://github.com/tseemann/snippy). Alignment was then focused on regions of the genome common to all isolates (the “core genome”) using the Snippy-core program, thus eliminating the complicating factors of insertions and deletions. Aligned sequences were then used to construct a maximum likelihood phylogenetic tree using RAxML utilising the GTRCAT model of rate heterogeneity and the software's autoMR and rapid bootstrap to find the best-scoring maximum likelihood tree and including tree branch lengths, defined as the number of base substitutions per site compared [32,33]. Finally, phylogenetic trees were illustrated using the web-based Microreact program [34].

## 3 Results and discussion

### 3.1 Risk factors for carriage of ABR E. coli in dogs at 16 weeks of age

In total, 223 16-week-old dogs were included in the analysis. For each of the 223 faecal samples provided, ABR *E. coli* carriage status was categorised as positive or negative for resistance to five test antibacterials: amoxicillin, cefalexin, ciprofloxacin, streptomycin or tetracycline, as set out in Materials and Methods. In a preliminary Chi-squared analysis, the only significant risk factor identified for 16-week-old dogs providing faecal samples carrying *E. coli* resistant to at least one antibacterial was having been fed raw meat (*p* < 0.001; Table 1). Subsequent univariable and multivariable logistic regression analyses showed a strong association between raw feeding and carriage of *E. coli* resistant to any one of the five antibacterials tested as well as individually with resistance to each of the antibacterials tested except cefalexin (Table 2).

**Table 2:** Univariable and multivariable logistic regression analyses using questionnaire data and antibacterial-resistant *E. coli* data for 16-week-old dogs (*n* = 223). Presentation: Odds ratio (95% confidence interval), *p-*value. Only risk actors significantly associated with resistance (*p-*value <0.05) are included.

Risk factor	Univariable (*n* = 223)	Multivariable for all samples (*n* = 223)
**Resistance to at least one antibacterial (*n*** **=** **108)**		
Fed raw food	3.98 (1.89 to 8.40) <0.001	3.98 (1.89 to 8.40) <0.001

**Resistance to ciprofloxacin (*n*** **=** **26)**		
Fed raw food	12.42 (5.01 to 30.78) <0.001	12.42 (5.01 to 30.78) <0.001

**Resistance to tetracycline (*n*** **=** **81)**		
Fed raw food	4.47 (2.21 to 9.05) <0.001	4.47 (2.21 to 9.05) <0.001

**Resistance to amoxicillin (*n*** **=** **93)**		
Fed raw food	3.30 (1.64 to 6.63) 0.001	3.18 (1.57 to 6.42) 0.001
Swam/paddled/ played in pond water	2.01 (1.12 to 3.61) 0.02	1.91 (1.05 to 3.48) 0.04

**Resistance to ce**f**alexin (*n*** **=** **34)**		
No statistically significant risk factors identified		

**Resistance to streptomycin (*n*** **=** **51)**		
Fed raw food	8.23 (3.95 to 17.15) <0.001	8.23 (3.95 to 17.15) <0.001

The most substantial AMR carriage risk associated raw feeding in 16-week-old dogs was carriage of FQ-R *E. coli* (Table 2), and fluoroquinolones are classed as critically important antibacterial drugs. This association has previously been reported in adult dogs in the UK; a study based on 445 dogs found that feeding raw poultry significantly increased the risk of carrying FQ-R *E. coli* in faeces [21]. Findings from the present study extend these earlier studies to show that the impact of raw feeding on ABR *E. coli* carriage can be seen as early as 10 weeks after the first introduction of solid food.

Antibacterial therapy might be expected to select for carriage of resistant *E. coli*, and we cannot entirely exclude the possibility that a few study dogs received antibacterials prior to sampling. However, the very young age of these dogs, coupled with our exclusion of dogs that had been hospitalised (and the fact that fluoroquinolones and tetracycline are not indicated for treatment of young dogs in the UK, and streptomycin is not used to treat dogs of any age in the UK) make antibacterial therapy an extremely unlikely undetected risk factor in this cohort.

### 3.2 Molecular epidemiology of FQ-R E. coli from dogs at 16 weeks of age

From twenty-six 16-week-old dogs that produced samples carrying FQ-R *E. coli*, 36 unique isolates were subjected to WGS (Table 3). Plasmid-mediated quinolone resistance mechanisms (PMQR) were found in only 3/36 FQ-R isolates, and in only one ST58 isolate carrying *qnrS*1 and a single *gyrA* mutation (Table 3) was there any suggestion that a PMQR was necessary for conferring FQ-R. The other two were an ST1196 isolate carrying *qnrS*1 and an ST1431 isolate carrying *qnrB*4, but in both there were also two mutations in *gyrA* and one in *parC*, sufficient to confer FQ-R in the absence of a PMQR gene [35]. Indeed, many of the FQ-R isolates collected in this study carried identical mutations and no PMQR genes (Table 3). Of the FQ-R isolates sequenced, ST744 (11/36 isolates) dominated, with 6/36 isolates identified as ST162 (Table 3). Others have reported carriage of CTX-M or AmpC-producing third generation cephalosporin-resistant (3GC-R) *E. coli* in dogs, and prevalence varies from study to study [[36], [37], [38]]. In our study we did not select for 3GC-R *E. coli* directly from faecal samples, so it is not possible for us to directly compare. However, of the 36 FQ-R isolates sequenced, we identified 3GC-R mechanisms in seven isolates. Three produced CTX-M-1, one CTX-M-15, one CTX-M-65, one DHA-1 (plasmid AmpC) and one hyper-produced AmpC due to chromosomal mutation (Table 3).

**Table 3:** Characterisation of FQ-R *E. coli* from 16-week-old dogs using WGS. Stars denote locally recruited dogs. Bold underlining denotes dogs fed raw food. 3GC-R mechanisms, where identified in WGS data, are noted.

Dog ID	*E. coli* ST	FQ-R mechanism(s)	3GC-R mechanism
DOG 3**	ST2179	*gyrA* S83L; *parC* S80I	CTX-M-65
DOG 4	ST744	*gyrA* S83L; *gyrA* D87N; *parC* A56T; *parC* S80I	CTX-M-1
**DOG 9**	ST162	*gyrA* S83L; *gyrA* D87N; *parC* S80I	
DOG 10	ST224	*gyrA* S83L; *gyrA* D87N; *parC* S80I	
DOG 11	ST1196	*gyrA* S83L; *gyrA* D87N; *parC* S80I; *qnrB4*	DHA-1
DOG 14	ST162	*gyrA* S83L; *gyrA* D87N; *parC* S80I	
**DOG 16**	ST744	*gyrA* S83L; *gyrA* D87N; *parC* A56T; *parC* S80I	
**DOG 17**	ST453	*gyrA* S83L; *gyrA* D87N; *parC* S80I	
**DOG 17**	ST58	*gyrA* S83L; *qnrS1*	
**DOG 17**	ST744	*gyrA* S83L; *gyrA* D87N; *parC* A56T; *parC* S80I	
**DOG 18**	ST224	*gyrA* S83L; *gyrA* D87N; *parC* S80I	CTX-M-1
**DOG 18**	ST744	*gyrA* S83L; *gyrA* D87N; *parC* A56T; *parC* S80I	
**DOG 19**	ST744	*gyrA* S83L; *gyrA* D87N; *parC* A56T; *parC* S80I	
**DOG 20**	ST162	*gyrA* S83L; *gyrA* D87N; *parC* S80I	
DOG 21**	ST10	*gyrA* S83L; *gyrA* D87N; *parC* S80I	
DOG 23	ST744	*gyrA* S83L; *gyrA* D87N; *parC* A56T; *parC* S80I	CTX-M-1
**DOG 24**	ST744	*gyrA* S83L; *gyrA* D87N; *parC* A56T; *parC* S80I	
**DOG 26**	ST1196	*gyrA* S83L; *gyrA* D87N; *parC* S80I	
**DOG 26**	ST1011	*gyrA* S83L; *gyrA* D87N; *parC* S80I	
**DOG 27**	ST1431	*gyrA* S83L; *gyrA* D87N; *parC* S80I; *qnrS1*	*ampC* -42C > T
DOG 29	ST4988	*gyrA* S83L; *gyrA* D87N*; parC* S80I	CTX-M-15
**DOG 30**	ST744	*gyrA* S83L; *gyrA* D87N; *parC* A56T; *parC* S80I	
DOG 31**	ST744	*gyrA* S83L; *gyrA* D87N; *parC* A56T; *parC* S80I	
DOG 32**	ST162	*gyrA* S83L; *gyrA* D87N; *parC* S80I	
DOG 33	ST542	*gyrA* S83L; *parC* S80I	
**DOG 34**	ST1011	*gyrA* S83L; *gyrA* D87N; *parC* S80I	
**DOG 34**	ST6817	*gyrA* S83L; *gyrA* D87N; *parC* S80I	
DOG 35	ST1011	*gyrA* S83L; *gyrA* D87N; *parC* S80I	
DOG 35	ST224	*gyrA* S83L; *gyrA* D87N; *parC* S80I	
**DOG 36**	ST155	*gyrA* S83L; *gyrA* D87N; *parC* S80I	
DOG 37	ST744	*gyrA* S83L; *gyrA* D87N; *parC* A56T; *parC* S80I	
DOG 37	ST162	*gyrA* S83L; *gyrA* D87N; *parC* S80I	
DOG 38**	ST744	*gyrA* S83L; *gyrA* D87N; *parC* A56T; *parC* S80I	
DOG 39**	ST162	*gyrA* S83L; *gyrA* D87N; *parC* S80I	
DOG 40	ST1193	gyrA S83L; gyrA D87N; parC S80I; *parE* L416F	
DOG 41**	ST1011	*gyrA* S83L; *gyrA* D87N; *parC* S80I	

### 3.3 Evidence of phylogenetic overlap between faecal FQ-R E. coli in puppies and those causing UTI in humans in the same geographical area

**Fig. 1:**
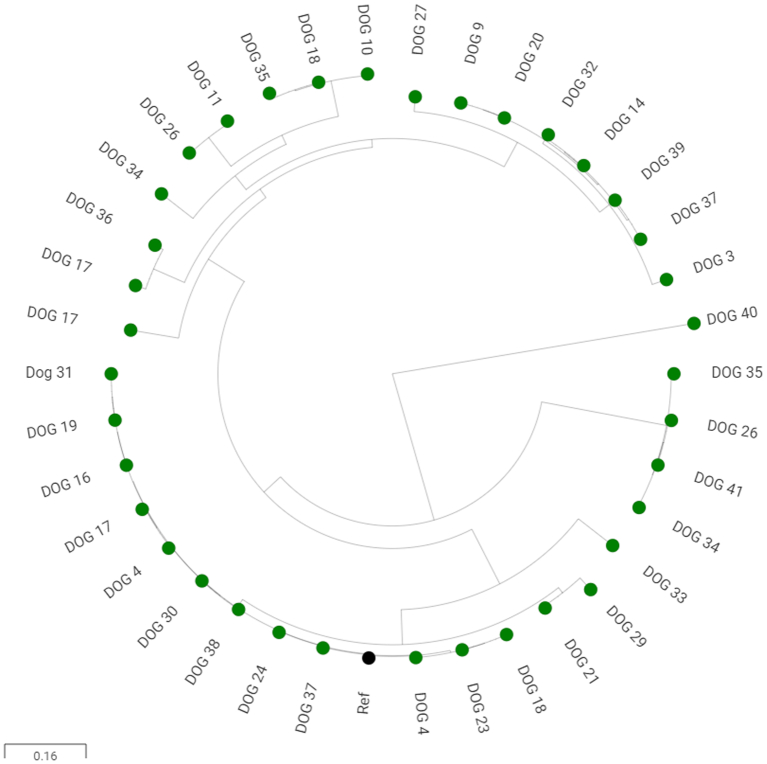
Core genome phylogenetic analysis of FQ-R *E. coli* from 16-week-old dogs. The randomly assigned Dog ID relevant to each isolate is labelled and links with Table 3. The reference sequence is represented by a black circle. FQ-R *E. coli* from dogs are represented by green circles. (For interpretation of the references to colour in this figure legend, the reader is referred to the web version of this article.)

A phylogenetic analysis of all the FQ-R isolates from puppies subjected to WGS in this study was constructed (Fig. 1). There were two clusters of isolates: ST162 and ST744 with multiple gyrase and topoisomerase mutations. Notably, one FQ-R isolate was ST1193, which is an important clone currently emerging in human infections and of the most pathogenic phylogroup, B2 [39]. It was therefore interesting to test relationships between FQ-R isolates from locally recruited dogs with human urinary FQ-R isolates from people living in the same geographical area as the locally recruited dogs.

Of the seven FQ-R isolates from locally recruited puppies (Table 3), five were of STs found amongst 188 FQ-R urinary *E. coli* from people living in the same 50 × 50 km geographical area, isolated within six months of collection of the isolates from puppies [8]. Of the canine isolates, one was ST10 and two each were ST744 and ST162 (Table 3). A SNP analysis based on core genome alignment of the ST744 isolates showed that one of the ST744 canine isolates was 47 SNPs different from a human urinary isolate. The closest SNP distance between a pair of ST744 human urinary *E. coli* in the same analysis was 931. SNP distances of <100 have previously been used to indicate potential sharing of isolates between different one-health compartments [8,40].

## 4 Conclusions

This study has identified raw meat feeding as a risk factor for the excretion of ABR *E. coli* in the faeces of 16-week-old puppies, with particularly strong impact on excretion of isolates resistant to the critically important fluoroquinolones. If owners insist on feeding raw meat to their dog, it is essential that they fully understand this practice puts their dog at risk of becoming colonised with bacteria resistant to critically important antibacterials. Notably, of 43 dogs fed raw meat, 34 of these (according to the owner survey) were fed what was described as “commercial raw food”, with the rest being fed meat not specifically marketed as dog food. Of these, 14/34 dogs fed commercial raw food were colonised with FQ-R *E. coli*, which is a similarly significant association (chi-square 33.9; *p* < 0.00001) to that seen for raw meat feeding overall (chi-square 40.2; *p* < 0.00001). Hence, it appears that feeding raw meat marketed as pet food is almost equally likely to result in colonisation with *E. coli* resistant to critically important antibacterials.

*E. coli* is the most clinically important opportunistic human bacterial pathogen [3]. ABR *E. coli* infections are more difficult to treat, and result in more morbidity and higher mortality rates [3]; there is also strong evidence that domestic pet dogs transmit ABR bacteria to humans [[9], [10], [11], [12]] and this study provides clear evidence of the faecal carriage within puppies of FQ-R *E. coli* clonally related to those that have also caused urinary infections in humans living in the same geographical region, collected within months of each other. This is suggestive of sharing between humans and dogs. Therefore, if owners feed raw meat to their dog, including that commercially marketed as pet food, practices that mitigate the risk of onward transmission of ABR *E. coli* – which are more likely to be carried by these dogs – to humans should be encouraged. These include strict hygiene practices when anyone (particularly those vulnerable to bacterial infection) interacts with a raw-fed dog along with scrupulous disposal of the dog's faeces so that they cannot pose a risk to the general human population by contaminating the wider environment with ABR *E. coli*.

## Funding

This work was funded by grant NE/N01961X/1 to M.B.A., T.A.C., K.M.T. and K.K.R. from the Antimicrobial Resistance Cross Council Initiative supported by the seven United Kingdom research councils. Dogs Trust fund the Generation Pup project.

## Author contributions

Conceived the Study: K.K.R., M.B.A.

Collection of Data: K.W. O.M., J.F., K.M. supervised by T.A.C., M.B.A., K.K.R.

Cleaning and Analysis of Data: O.M. K.W. A.H., V.C.G., supervised by M.B.A., K.M.T., K.K.R.

Initial Drafting of Manuscript: K.W., O.M., K.K.R., M.B.A.

Corrected and Approved Manuscript: All Authors.

## Declaration of Competing Interest

M.B.A. is married to the owner of a veterinary practice that sells various mass-manufactured dog foods amounting to a value less than 5% of total turnover. Otherwise, none of the above named authors is declaring any conflict of interest in relation to the above titled submitted manuscript.
